# Diffusion-weighted and dynamic contrast-enhanced MRI of pancreatic adenocarcinoma xenografts: associations with tumor differentiation and collagen content

**DOI:** 10.1186/s12967-016-0920-y

**Published:** 2016-06-07

**Authors:** Catherine S. Wegner, Jon-Vidar Gaustad, Lise Mari K. Andersen, Trude G. Simonsen, Einar K. Rofstad

**Affiliations:** Group of Radiation Biology and Tumor Physiology, Department of Radiation Biology, Institute for Cancer Research, Norwegian Radium Hospital, Oslo University Hospital, Box 4953, Nydalen, 0424 Oslo, Norway

**Keywords:** Pancreatic ductal adenocarcinoma, Magnetic resonance imaging, Parametric MR images, Differentiation of PDAC, Stroma of PDAC

## Abstract

**Purpose:**

The aggressiveness of pancreatic ductal adenocarcinoma (PDAC) is highly dependent on the level of differentiation and the composition of the stroma. In this preclinical study, we investigated the potential of diffusion-weighted magnetic resonance imaging (DW-MRI) and dynamic contrast-enhanced magnetic resonance imaging (DCE-MRI) as noninvasive methods for providing information on the differentiation and the stroma of PDACs.

**Methods:**

Xenografted tumors initiated from four PDAC cell lines (BxPC-3, Capan-2, MIAPaCa-2, and Panc-1) were included in the study. DW-MRI and DCE-MRI were carried out on a 7.05-T MR scanner, and tumor images of ADC (the apparent diffusion coefficient), *K*^trans^ (the volume transfer constant of Gd-DOTA), and *v*_e_ (the fractional distribution volume of Gd-DOTA) were produced. The level of differentiation and the amount and structure of collagen I and collagen IV were determined by examining histological preparations.

**Results:**

Differentiated tumors showed lower levels of collagen I and collagen IV than non-differentiated tumors. Significant correlations were found between ADC and *v*_e_, and both parameters differentiated clearly between collagen-rich non-differentiated tumors and differentiated tumors containing less collagen.

**Conclusion:**

Differentiated PDAC xenografts show higher ADC values and higher *v*_e_ values than their non-differentiated counterparts. This observation supports the application of parametric MR images as tumor biomarkers in PDAC. Patients showing low values of ADC and *v*_e_ most likely have non-differentiated tumors with extensive stroma and, hence, poor prognosis.

**Electronic supplementary material:**

The online version of this article (doi:10.1186/s12967-016-0920-y) contains supplementary material, which is available to authorized users.

## Background

Pancreatic ductal adenocarcinoma (PDAC) is a highly aggressive disease with poor prognosis. The majority of the patients are subjected to chemotherapy, which only marginally increases their lifespan [1]. Aggressive growth and treatment resistance are associated with poor differentiation and extensive tumor stroma in PDAC [1–4].

The stromal component is a defining characteristic of PDAC, constituting the majority of the tumor volume [5]. In addition to fibroblasts and immune cells, it is characterized by an often dense extracellular matrix [6]. Stromal fibroblasts promote local invasion [4, 7], and several components of the extracellular matrix have been shown to decrease the cytotoxicity of anticancer drugs in pancreatic cancer cell lines [8]. The stroma has also been postulated to serve as a mechanistic barrier to chemotherapy [9]. Collagen is a major component of the stroma, and collagen I is highly up-regulated in PDAC [10]. Both collagen I and IV have been shown to have prognostic power, and high levels of these matrix components are associated with poor survival rates [11, 12].

The composition of the stroma is associated with the level of differentiation in PDAC, and patients with poorly differentiated tumors have worse prognosis than those with well differentiated tumors [13]. Radiological studies attempting to use diffusion-weighted magnetic resonance imaging (DW-MRI) or dynamic contrast-enhanced magnetic resonance imaging (DCE-MRI) to distinguish between well and poorly differentiated PDACs have given conflicting results [14–19]. Highly different protocols were used for image acquisition and analysis in these investigations, and consequently, comparisons of observations are difficult. Moreover, these investigations have severe limitations. First, the MRI was carried out under suboptimal conditions in some investigations, and other investigations suffer from inadequate image analysis. Second, DW-MRI and DCE-MRI were not performed on the same patient cohorts, and the collagen component of the imaged tissue was not assessed.

MRI can be carried out under well-controlled conditions in preclinical studies, and the tumor tissue can be excised in its entirety immediately after the imaging for histological examination. Experimental tumors should therefore be useful for investigating whether DW-MRI and DCE-MRI, either separately or in combination, may have the power to distinguish between poorly and well differentiated PDACs. The possibility that DW-MRI and DCE-MRI can provide images reflecting the histological appearance of PDACs was examined in detail in the preclinical study reported here. By subjecting PDAC xenografts to DW-MRI, DCE-MRI, and detailed histological examinations, we provide strong evidence that parametric images derived from DW-MRI and DCE-MRI data can distinguish collagen-rich non-differentiated tumors from differentiated tumors with less collagen.

## Methods

### Tumor models

BxPC-3, Capan-2, MIAPaCa-2, and Panc-1 (American Type Culture Collection, VA, USA) human PDAC xenografts grown in adult (8–12 weeks of age) female BALB/c *nu*/*nu* mice were used as preclinical tumor models. Tumors were initiated from cells cultured in RPMI-1640 (25 mmol/l HEPES and l-glutamine) medium supplemented with 13 % bovine calf serum, 250 mg/l penicillin, and 50 mg/l streptomycin. Approximately 2.5 × 10^6^ cells in 20–30 μl of Hanks’ balanced salt solution were inoculated in the gastrocnemius muscle. Tumors with volumes of 400–1200 mm^3^ were included in the study. Tumor volume was determined from MR images, and tumor volume doubling time (*T*_d_) was calculated as ${{T_{\text{d}} = t \times {\text{ln2}}} \mathord{\left/ {\vphantom {{T_{\text{d}} = t \times {\text{ln2}}} {\left( {{ \ln }V_{\text{t}} - { \ln }V_{0} } \right)}}} \right. \kern-0pt} {\left( {{ \ln }V_{\text{t}} - { \ln }V_{0} } \right)}}$, where *t* = time from tumor cell inoculation to MR scanning, *V*_t_ = tumor volume at time of scanning, and *V*_0_ = tumor volume at time of cell inoculation (set to 1 mm^3^). The tumors of all models showed only negligible regions with necrotic, cystic, or hemorrhagic tissue. The animal experiments were approved by the Institutional Committee on Research Animal Care and were performed according to the Interdisciplinary principles and guidelines for the use of animals in research, marketing, and education (New York Academy of Sciences, New York, NY, USA).

### Magnetic resonance imaging

MRI was carried out at the MRI Core Facility for Preclinical Cancer Research, Oslo University Hospital, by using a Bruker Biospec 7.05-T bore magnet and a mouse quadrature volume coil (Bruker Biospin, Ettlingen, Germany). Twelve BxPC-3, 12 Capan-2, 12 MIAPaCa-2, and 16 Panc-1 tumors were subjected to MRI, and DCE-MRI was carried out immediately after DW-MRI. The tumors were positioned in the isocenter of the magnet and were imaged with 7–8 axial slices covering the entire volume. The mice were given gas anesthesia (~4.0 % Sevofluran in O_2_; Baxter, IL, USA) at a flow rate of 0.5 l/min during imaging. Respiration rate and body core temperature were monitored continuously by using an abdominal pressure sensitive probe and a rectal temperature probe (Small Animal Instruments, New York, NY, USA). The body core temperature was kept at 37 °C by automated hot air flow regulation, and the gas anesthesia was adjusted manually to maintain a stable respiration rate. Anatomical *T*_2_-weighted images were obtained prior to DW-MRI and DCE-MRI by using a fast spin echo pulse sequence (RARE) with a repetition time (TR) of 2500 ms, an echo time (TE) of 35 ms, an image matrix of 128 × 128, a field of view (FOV) of 3 × 3 cm^2^, a slice thickness of 0.7 mm, a slice gap of 0.3 mm, 2 averages, and fat suppression.

DW-MRI was carried out as described previously [20]. Briefly, we applied a diffusion-weighted single-shot fast spin echo pulse sequence (RARE) with a TR of 1300 ms, a TE of 26 ms, an image matrix of 64 × 64, a FOV of 3 × 3 cm^2^, a slice thickness of 0.7 mm, a slice gap of 0.3 mm, and fat suppression. Four diffusion-weightings with diffusion encoding constants (*b*) of 200, 400, 700, and 1000 s/mm^2^, a diffusion gradient duration of 7 ms, and a diffusion separation time of 14 ms were used. Values of *b* ranging from 200 to 1000 s/mm^2^ were chosen to avoid perfusion effects [21, 22]. Diffusion sensitization gradients were applied in three orthogonal directions, and ADC values were calculated for each direction by using in-house-made software developed in Matlab (MathWorks, Natick, MA, USA). Furthermore, the directional diffusion images were averaged on a voxel-by-voxel basis to non-directional diffusion images, and these non-directional images were used to calculate ADC maps. As expected for isotropic tumors, the ADC values were found to be independent of the direction of the diffusion sensitization gradients (Additional file 1: Figure S1).

**Fig. 1:**
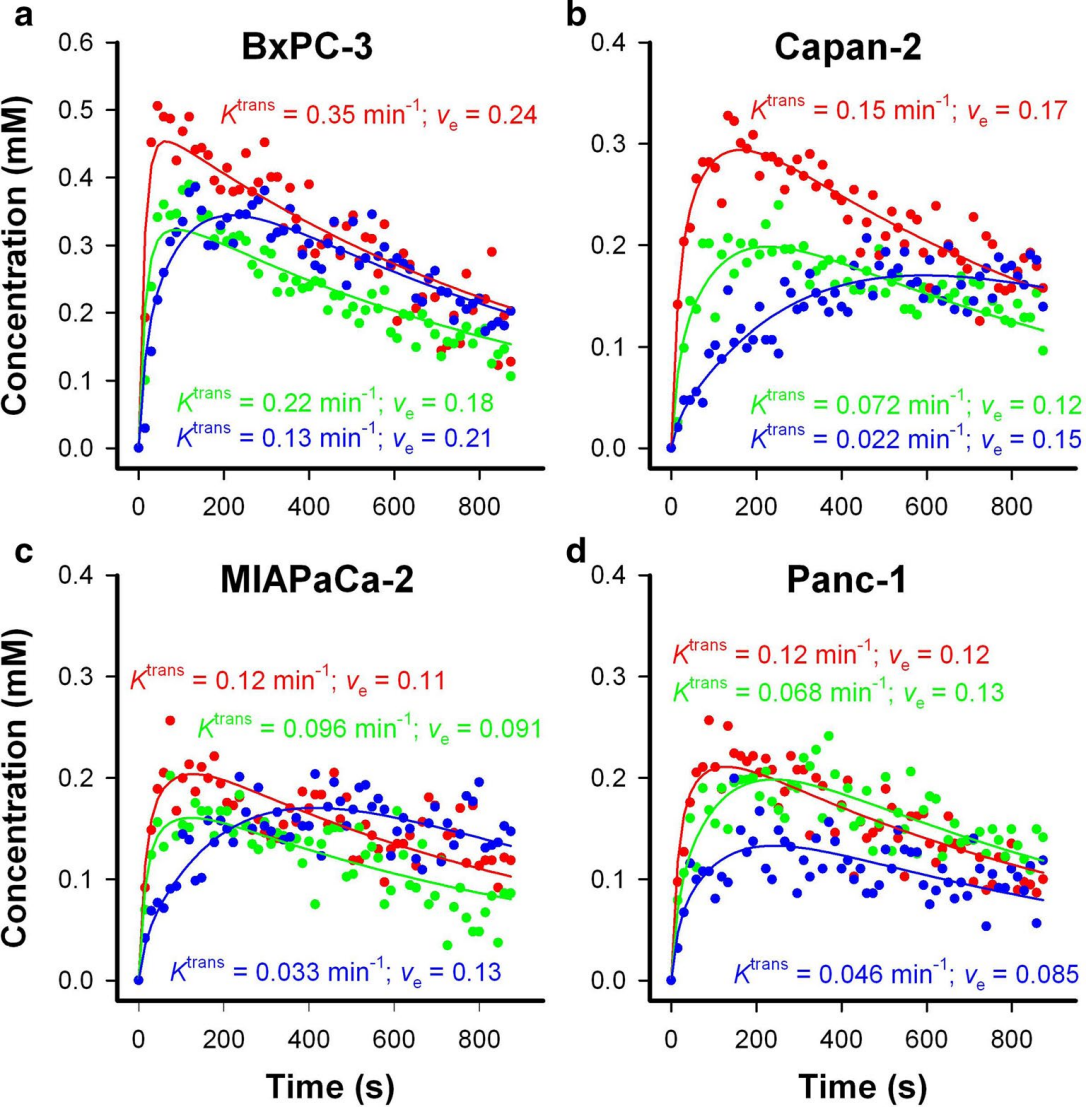
Pharmacokinetic analysis of representative tumors. *Plots* of Gd-DOTA concentration versus time (*symbols*) and the corresponding curve fits (*solid* *lines*) for single voxels of a BxPC-3 (**a**), Capan-2 (**b**), MIAPaCa-2 (**c**), and Panc-1 (**d**) tumor. The *K* ^trans^ and *v*_e_ values of the voxels were determined by the Tofts pharmacokinetic model, and are shown in the panels in the *same* *color* as the corresponding *curve*

DCE-MRI with Gd-DOTA (Dotarem, Guerbet, Paris, France) as contrast agent was performed as described earlier [20]. Briefly, a fast spin echo pulse sequence (RARE) with TRs of 200, 400, 800, 1500, 3000, and 5000 ms, a TE of 8.5 ms, an image matrix of 128 × 128, a FOV of 3 × 3 cm^2^, a slice thickness of 0.7 mm, and a slice gap of 0.3 mm was applied to measure precontrast *T*_1_-values (*T*_10_-map). Gd-DOTA was diluted to a concen-tration of 0.06 M and administered in the tail vein in a bolus dose of 5.0 ml/kg body weight during a period of 5 s by using an automated infusion pump (Harvard Ap-paratus, Holliston, MA, USA). A three-dimensional SPGR pulse sequence (3D-FLASH) with a TR of 10 ms, a TE of 2.07 ms, a flip angle (α) of 20°, an image matrix of 128 × 128, and a FOV of 3 × 3 cm^2^ was used to produce dynamic *T*_1_-weighted images at a temporal resolution of 14.8 s. Numerical values of the volume transfer constant (*K*^trans^) and the fractional distribution volume (*v*_e_) of the contrast agent were determined on a voxel-by-voxel basis by using the Tofts pharmacokinetic model [23]. Calculation of Gd-DOTA concentrations and pharmacokinetic modeling were done with in-house-made software developed in Matlab (MathWorks). Representative plots of Gd-DOTA concentration versus time and the corresponding curve fits are presented in Fig. 1, illustrating that good fits were obtained for single voxels with highly different uptake of Gd-DOTA. The intratumor heterogeneity in *K*^trans^ and *v*_e_ was substantial, and to ensure that our DCE-MRI method provided high-quality *K*^trans^ and *v*_e_ images, it was verified that structural tumor elements were clearly recognizable in parametric images of ad-jacent slices (Additional file 1: Figure S2).

**Fig. 2:**
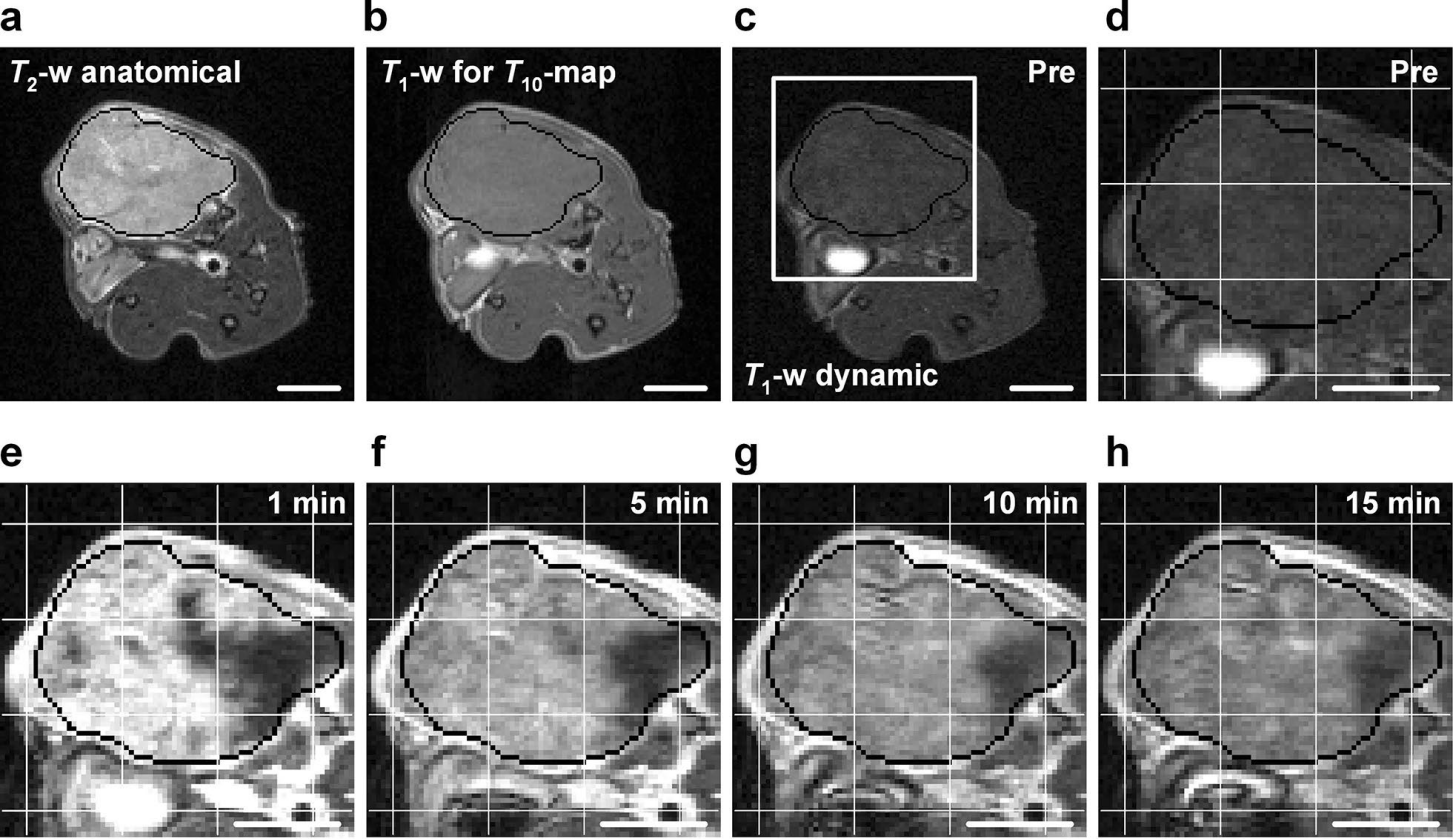
MR images of a representative tumor. *T*_2_-weighted anatomical image (**a**) and *T*_1_-weighted images (**b**–**h**) from an experiment with a BxPC-3 tumor. The *T*_1_-weighted images were recorded with a fast spin echo pulse sequence for calculation of precontrast *T*_1_ values (**b**) or with a three-dimensional SPGR sequence for acquiring dynamic series (**c**–**h**). Dynamic images were recorded before and every 14.8 s after contrast administration. The dynamic images in (**c**–**h**) refer to a precontrast image (**c**, **d**) and images recorded 1 min (**e**), 5 min (**f**), 10 min (**g**), and 15 min (**h**) after contrast administration. The region within the *square* in (**c**) is *highlighted* in (**d**–**h**). Tumor regions of interest (ROI) were depicted in the anatomical image (**a**) and were transferred to the other images since the different pulse sequences were performed with identical geometrical settings. The tumor ROI is outlined with *black* in the images (**a**–**h**). The coordinates of the tumor voxels are defined by the *white* *grids* (one line per 20 voxels) in (**d**–**h**). The images illustrate that the tumor did not move or change shape during the MRI. *Scale* *bars* 5 mm

The analysis of the DW-MRI and DCE-MRI data was based the entire volume of the tumors without including peritumoral blood vessels. Regions of interest (ROIs) encompassing the tumor tissue were depicted in the *T*_2_-weighted anatomical images acquired prior to the DW-MRI, and these ROIs were transferred to the DW and DCE images (Fig. 2). The quantitative analyses were carried out on these ROIs without excluding any voxels. It was verified that the tumors did not move between imaging sequences or during imaging series (Fig. 2), and consequently, motion correction algorithms were not used.

### Histological examinations

Histological sections were cut from three different positions in each tumor and stained with hematoxylin and eosin (HE) by using a standard procedure or immunostained for connective tissue by using a peroxidase-based indirect staining method [24]. An anti-collagen I rabbit polyclonal antibody (ab34710, Abcam, Cambridge, UK) or an anti-collagen IV rabbit polyclonal antibody (ab6586, Abcam) was used as primary antibody. Diaminobenzidine was used as chromogen, and hematoxylin was used for counterstaining. Tumor differentiation was determined from HE stained sections and classified on a scale from 1 to 4, where 1 refers to non-differentiated tumors and 4 refers to well differentiated tumors. The extent of collagen I and collagen IV staining was assessed semiquantitatively by using a scale from 0 to 4, where 0 and 4 represent no staining and maximal staining, respectively.

### Statistical analysis

The Pearson product moment correlation test was used to search for correlations between parameters. Statistical comparisons of data sets were carried out by using the Student’s *t* test when the data sets complied with the conditions of normality and equal variance. Under other conditions, comparisons were carried out by nonparametric analysis using the Mann–Whitney rank-sum test. Probability values of *P* < 0.05, determined from two-sided tests, were considered significant. The SigmaStat statistical software (SPSS Science, Chicago, IL, USA) was used for the statistical analysis.

## Results

**Fig. 3:**
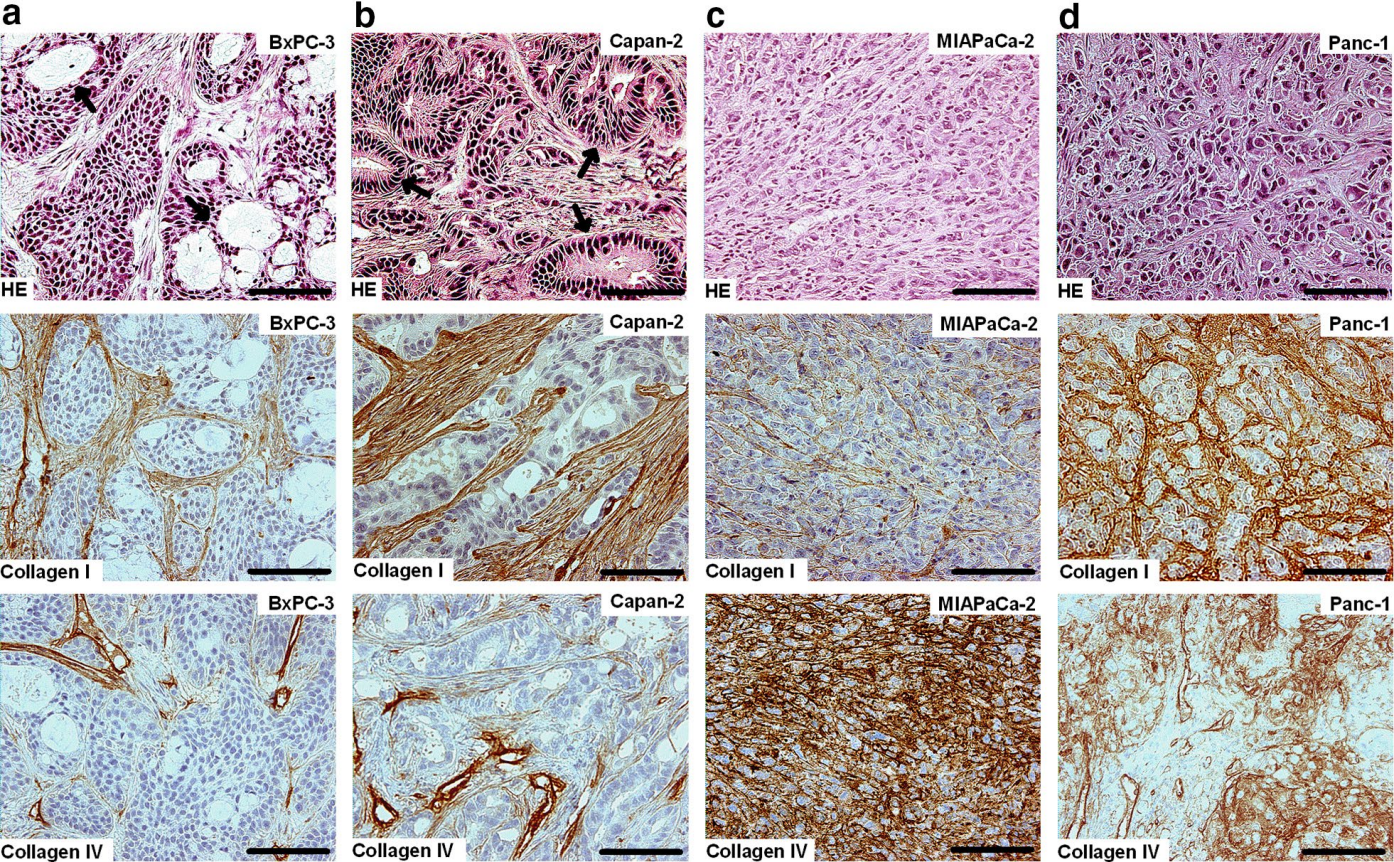
Differentiated and non-differentiated PDAC tumor models. Histological preparations of a BxPC-3 (**a**), Capan-2 (**b**), MIAPaCa-2 (**c**), and Panc-1 (**d**) tumor. *Arrows* point to ductal structures seen in the two differentiated models, BxPC-3 and Capan-2. The non-differentiated models MIAPaCa-2 and Panc-1 did not show ductal structures. *Scale* *bars* 100 µm. *Upper* *panels* HE staining. *Middle* *panels* Collagen I staining. *Lower* *panels* Collagen IV staining

The four PDAC tumor models differed considerably in histological appearance. The BxPC-3 and Capan-2 models were differentiated and showed distinct ductal structures (Fig. 3a, b), whereas the MIAPaCa-2 and Panc-1 models were non-differentiated and showed an apparently random distribution of the tumor cells (Fig. 3c, d). The tumor models differed notably also in the amount and distribution of collagen I and collagen IV. In the differentiated models, collagen I and IV were found in thick filament bundles separating clusters of tumor cells (Fig. 3a, b), whereas the non-differentiated models showed a strikingly divergent collagen arrangement with thin fibers of collagen I and IV surrounding single tumor cells (Fig. 3c, d). On the other hand, the individual tumors of each PDAC model did not differ in differentiation or collagen staining. In fact, they could not be distinguished from each other, regardless of whether HE stained or immunostained sections were examined and regardless of whether the sections were prepared from central or peripheral regions of the tumors.

**Fig. 4:**
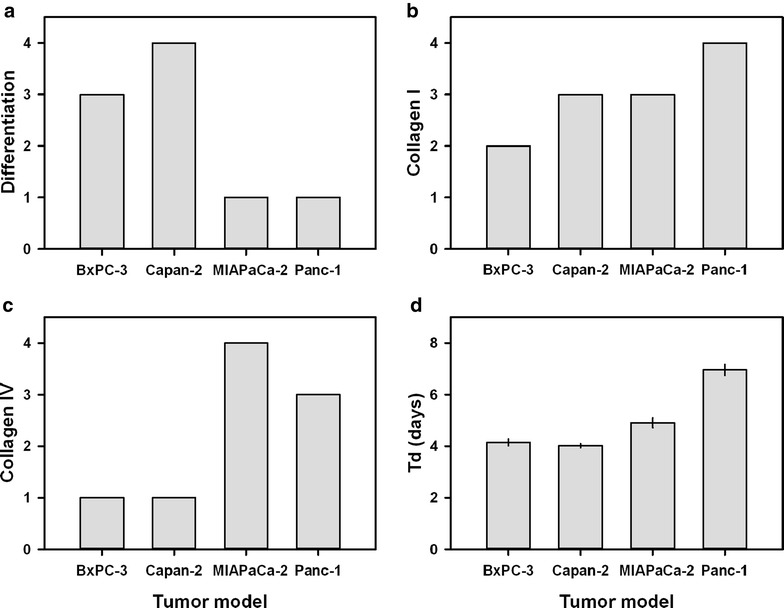
Differentiated PDAC tumor models have less collagen and grow faster than non-differentiated PDAC tumor models. Level of differentiation (**a**), amount of collagen I (**b**), amount of collagen IV (**c**), and volume doubling time (**d**) of BxPC-3, Capan-2, MIAPaCa-2, and Panc-1 tumors. The individual tumors of the same model were indistinguishable histologically in both HE stained and immunostained sections, but differed in growth rate. *Columns* and *bars* in (**d**) Mean ± SEM of 12–16 tumors

Semiquantitative analysis revealed that the two differentiated PDAC models showed only minor differences in collagen content even though BxPC-3 was clearly less differentiated than Capan-2, and furthermore, the difference in collagen content between the two non-differentiated PDAC models was also small (Fig. 4a, c). On the other hand, the content of collagen was clearly higher in the non-differentiated models than in the differentiated models, and the difference was particularly large for collagen IV. Interestingly, the volume doubling times of the MIAPaCa-2 and Panc-1 tumors were significantly longer than those of the BxPC-3 and Capan-2 tumors (Fig. 4d; *P* < 0.001); in other words, the tumors of the differentiated PDAC models grew faster than those of the non-differentiated models.

**Fig. 5:**
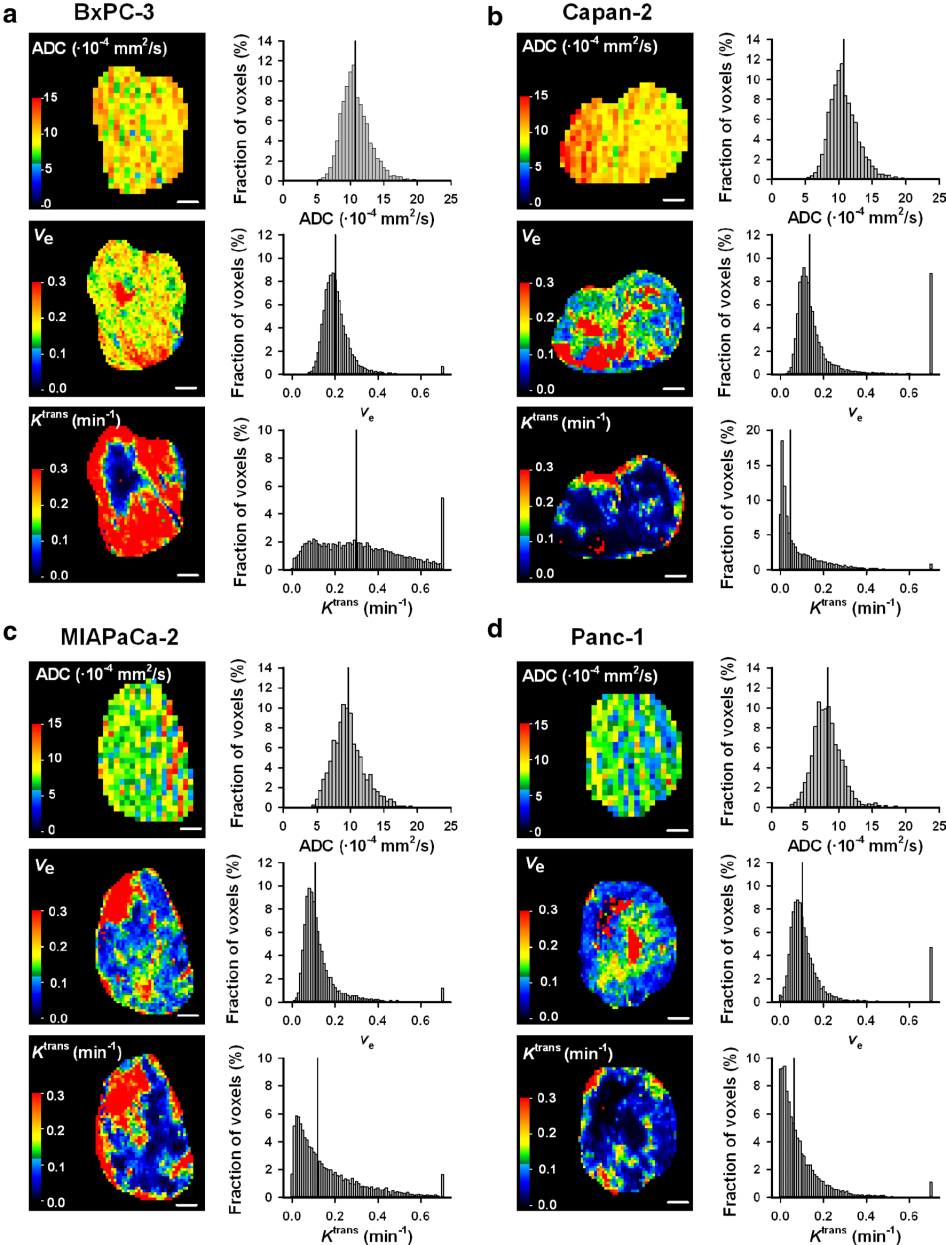
Images and frequency distributions of MR parameters of differentiated and non-differentiated PDAC tumor models. ADC, *v*_e_, and *K* ^trans^ images and frequency distributions of a representative BxPC-3 (**a**), Capan-2 (**b**), MIAPaCa-2 (**c**), and Panc-1 (**d**) tumor, illustrating that the intratumor heterogeneity in these MR parameters was considerable in the four PDAC models. The images refer to a central axial section of the tumors, whereas the frequency distributions are based on the individual voxel values of all tumor sections. *Color bars* ADC, *v*_e_, and *K* ^trans^ *scales*. *Scale bars* 2 mm. *Vertical lines* median values

ADC, *v*_e_, and *K*^trans^ images and frequency distributions of a representative tumor of each PDAC model are shown in Fig. 5. The intratumor heterogeneity in these MR parameters was substantial in all four PDAC models. In general, the tumors showed higher *K*^trans^ values in the periphery than in central regions, whereas the highest values of *v*_e_ and ADC were more randomly distributed within the tumor volume. This observation emphasizes the importance of analyzing the entire tumor volume, as was done here. Furthermore, Fig. 5 shows that the shape of the frequency distributions of ADC, *v*_e_, and *K*^trans^ differed among individual tumors. In some tumors, these parameters showed frequency distributions that were far from Gaussian, and consequently, the median value of each parameter rather than the mean value was calculated for each tumor and used in the further analysis.

**Fig. 6:**
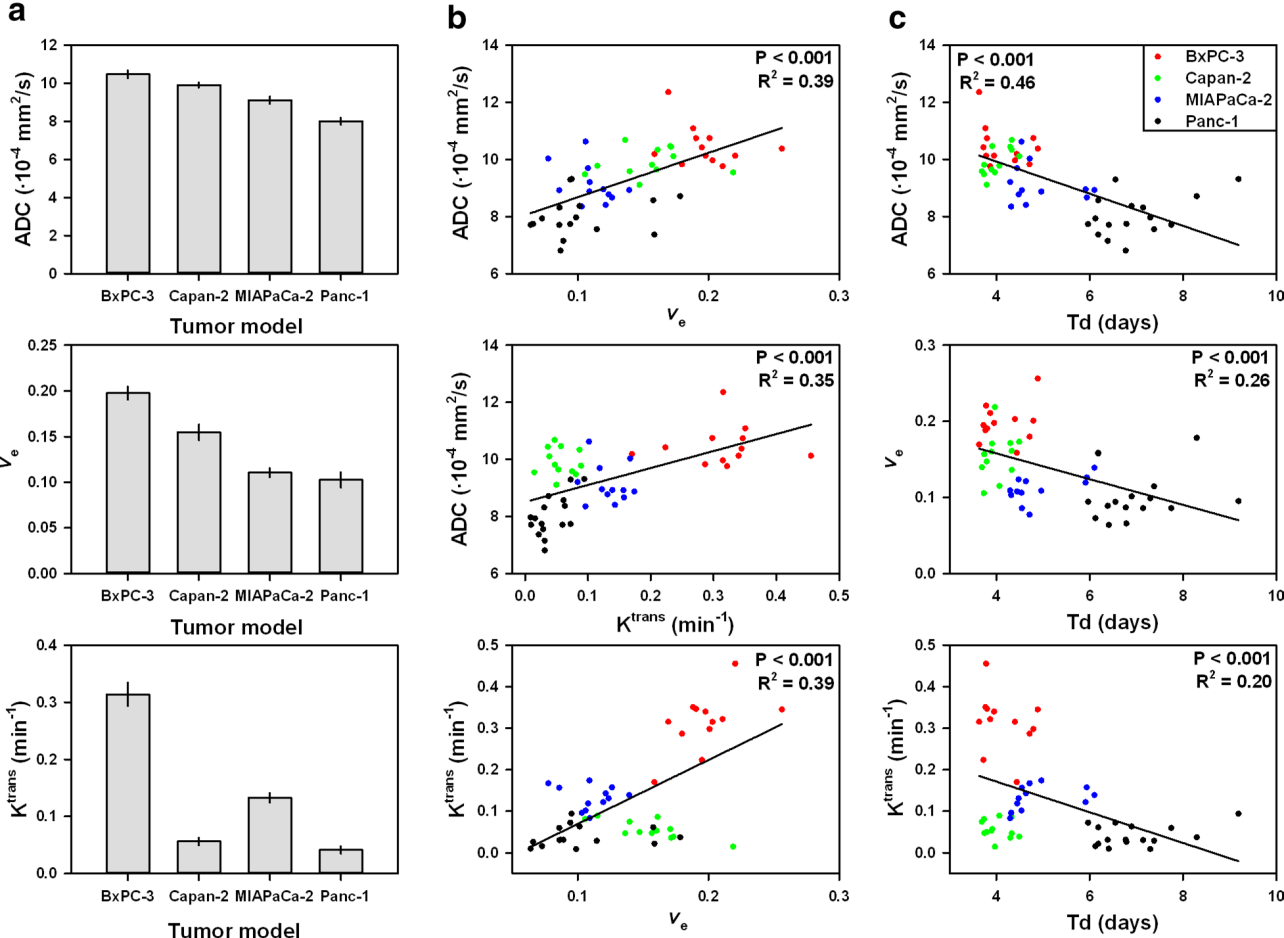
Relationships between ADC, *K* ^trans^, *v*_e_, and *T*_d_ of differentiated and non-differentiated PDAC tumor models. ADC, *v*_e_, and *K* ^trans^ (**a**), ADC *vs* *v*_e_, ADC *vs* *K* ^trans^, and *K* ^trans^ *vs* *v*_e_ (**b**), and ADC *vs* *T*_d_, *v*_e_ *vs* *T*_d_, and *K* ^trans^ *vs* *T*_d_ (**c**) for BxPC-3, Capan-2, MIAPaCa-2, and Panc-1 tumors. The relationships in (**b**) and (**c**) are statistically significant. *Columns* mean of the median value of 12–16 tumors. *Error bars* SEM. *Points* individual tumors. *Linear curves* regression lines

The MR parameters were found to differ significantly among the PDAC models (Fig. 6a). Compared with the non-differentiated models, the differentiated models showed higher values of ADC (*P* < 0.001) and higher values of *v*_e_ (*P* < 0.001), whereas the differences in *K*^trans^ were not associated with the level of differentiation. Furthermore, significant correlations were found between the different MR parameters (Fig. 6b). Thus, ADC was positively correlated to *v*_e_ (*P* < 0.001) and *K*^trans^ (*P* < 0.001), and *K*^trans^ was positively correlated to *v*_e_ (*P* < 0.001). Significant correlations were also found between the MR parameters and tumor growth rate (Fig. 6c); ADC, v_e_, and *K*^trans^ decreased with increasing tumor volume doubling time (*P* < 0.001 for all parameters).

## Discussion

The possibility that DW-MRI and/or DCE-MRI may be useful noninvasive methods for providing information on the level of differentiation in PDAC was investigated in this preclinical study by performing experiments with four xenograft models of PDAC. Preclinical investigations have the advantage to clinical investigations that several copies of the same patient’s tumor can be studied. The individual xenografted tumors of the same PDAC model were initiated from the same cell line in this study, and consequently, these tumors were genetically equal and represent a single human tumor. These tumors were for the same reason indistinguishable histologically, both in HE stained and immunostained preparations. However, individual tumors of the same model may differ somewhat in vascular density and blood perfusion for two main reasons. First, tumor vascularity decreases with increasing tumor size in most experimental tumors, primarily because the tumor parenchyma outgrows the vascular network [25]. Second, stochastic processes involved in tumor angiogenesis may lead to differences in vascular density and blood perfusion between individual tumors of the same model [26]. Therefore, median ADC, *K*^trans^, and *v*_e_ differed among the individual tumors of the same PDAC model in this study, but these differences were small compared with the differences between the four models. Human PDACs may show substantial intratumor heterogeneity in vascularity and, hence, in parametric MR images, and this intratumor heterogeneity may be captured in preclinical studies involving several tumors of the same model.

The four preclinical tumor models included in this study differed highly in structural appearance and represent two clinically important groups of PDAC, differentiated and non-differentiated tumors. In agreement with earlier findings, Capan-2 and BxPC-3 were shown to be differentiated, whereas MIAPaCa-2 and Panc-1 were found to be non-differentiated [27]. Moreover, the non-differentiated models were found to have more collagen I and collagen IV and a more densely packed matrix of collagen fibers than the differentiated models. Because both poor differentiation and a high level of collagen fibers are associated with poor prognosis in PDAC [11–13], xenografted BxPC-3, Capan-2, MIAPaCa-2, and Panc-1 tumors should be well-suited preclinical models for providing clinically relevant answers to the questions addressed in the present MRI study.

The DCE-MRI data were analyzed by using the Tofts pharmacokinetic model [23]. This model is based on several assumptions. The most important of these are that the interstitial concentration of contrast agent is uniform within the voxels, that the contribution of intravascular contrast agent to the total tumor concentration can be ignored, and that any effects of water exchange are insignificant [23]. When these assumptions are adequately fulfilled, *K*^trans^ is determined by the blood flow and the permeability of the vessel wall, and *v*_e_ is determined by the fractional distribution volume of the contrast agent in the tumor tissue [28]. The Tofts model gave good fits to our experimental data at the single voxel level in all four PDAC models, suggesting that this pharmacokinetic model is suitable for analyzing the DCE-MRI data recorded in this study. Other more complex pharma-cokinetic models are also being used frequently, including the Brix model [29] and the shuttle speed model [30]. Comparative studies of the usefulness of these models in the analysis of human DCE-MRI data have suggested that the Tofts model is preferable to the more complex pharmacokinetic models [31]. Furthermore, a consensus meeting involving MR scientists from several institutions has recommended the Tofts models for analysis of preclinical and clinical DCE-MRI series [28].

Our study showed that ADC was higher in the differentiated than in the non-differentiated PDAC models. Most likely, this difference was partly a consequence of the difference in the density of the extracellular collagen matrix and partly a consequence of the presence of ductal structures with central cavities in the differentiated tumors. ADC is a measure of water diffusion [32, 33] and has been shown to decrease with increasing cell density [34]. The low cell density associated with the ductal structures of the differentiated tumors probably restricted the diffusion of water to a lesser extent than the densely packed cells of the non-differentiated tumors, thus contributing to the differences seen in ADC. Furthermore, collagen has been shown to impede water diffusion [22, 35]. Because the collagen network of the differentiated tumors primarily surrounded clusters of tumor cells whereas that of the non-differentiated tumors primarily surrounded single cells, the collagen network may have restricted the diffusion of water to a lesser extent in the differentiated tumors than in the non-differentiated tumors, thus contributing to the observed differences in ADC.

Also *v*_e_ was found to be higher in the differentiated than in the non-differentiated PDAC models. This parameter is a measure of the distribution volume of the contrast agent and is determined by the fractional volume of the extravascular extracellular space and the density of the extracellular matrix [28]. Consequently, the differences between the differentiated and non-differentiated tumors in cell density and collagen content, particularly collagen IV, most likely contributed to the differences in *v*_e_ detected here.

The possibility that ADC may be associated with *v*_e_ has not been investigated in PDAC. However, several studies have attempted to show correlations between ADC and *v*_e_ in other types of cancer, but significant correlations have not been found in any study thus far [36–39]. In contrast, a significant correlation between ADC and *v*_e_ was detected in the present study, possibly because both ADC and *v*_e_ are strongly influenced by the extracellular matrix in PDAC xenografts. However, due to the nature of these MR parameters, it is likely that the distribution of collagen fibers has a stronger influence on ADC than *v*_e_. It should be noticed that the two non-differentiated tumor models both showed a more dispersed collagen matrix and a higher total amount of collagen than the two differentiated tumor models. Moreover, the non-differentiated tumor models grew more slowly than the differentiated ones, possibly because their growth rate was limited by the rate of collagen synthesis, and consequently, both ADC and *v*_e_ were found to decrease with increasing tumor volume doubling time.

Our study also showed that *K*^trans^ differed significantly among the four PDAC models, but in contrast to ADC and *v*_e_, this parameter did not differ between the differentiated and non-differentiated models. *K*^trans^ is a parameter that is influenced primarily by tumor blood perfusion and vessel wall permeability [28, 32]. Because BxPC-3 showed high *K*^trans^ values and Capan-2 showed low values of *K*^trans^, it is not likely that tumor perfusion plays a significant role in the differentiation of PDAC. Furthermore, significant correlations were found between *K*^trans^ on the one hand and ADC, *v*_e_, and *T*_d_ on the other. However, these correlations appeared primarily because the BxPC-3 tumors showed substantially higher *K*^trans^ values than the tumors of the other models, and the biological significance of these correlations may therefore be limited.

The study reported here has significant clinical implications. The potential of DW-MRI and DCE-MRI as methods for distinguishing between well and poorly differentiated PDACs has been addressed in several clinical studies, however, with conflicting results [17–19, 40]. Some studies have suggested that DCE-MRI may have prognostic power and thus may predict survival in PDAC patients, and furthermore, that DW-MRI may have the power to detect non-differentiated PDAC tumors with dense fibrosis [17, 40]. However, other studies have not shown similar relationships and thus do not support these suggestions [18, 19]. This discrepancy has raised doubt about the usefulness of parametric MR images in the diagnostics of PDAC; however, the discrepancy may be caused by several conditions, including low number of patients and inadequate scanning protocols (e.g., DW-MRI with only two *b* values) in some investigations. The present preclinical study indeed suggests that both DW-MRI and DCE-MRI have the power to discriminate between well differentiated and poorly differentiated tumors, and moreover, that these imaging methods may complement one another in the diagnostics of PDAC.

## Conclusions

Both DW-MRI and DCE-MRI have the power to discriminate between differentiated and non-differentiated PDAC xenografts, partly because the collagen content is higher in non-differentiated than in differentiated tumors. PDAC patients having developed non-differentiated and/or collagen-rich primary tumors have particularly poor prognosis, and it is likely that low ADC and low *v*_e_ values are characteristic features of the tumors of these patients. Consequently, DW-MRI and DCE-MRI are noninvasive imaging methods with an added joint potential that should be taken advantage of in the diagnostics of PDAC.

## Additional file

10.1186/s12967-016-0920-y ADC did not differ with the direction of the diffusion sensitization gradient. **Figure S2.** Structural elements were clearly recognizable in parametric images of adjacent tumor slices.

## References

[1] Neesse A, Algul H, Tuveson DA, Gress TM (2015). Stromal biology and therapy in pancreatic cancer: a changing paradigm. Gut.

[2] Ahn SJ, Park MS, Lee JD, Kang WJ (2014). Correlation between 18F-fluorodeoxy-glucose positron emission tomography and pathologic differentiation in pan-creatic cancer. Ann Nucl Med.

[3] Karademir S, Sokmen S, Terzi C, Sagol O, Ozer E, Astarcioglu H (2000). Tumor angiogenesis as a prognostic predictor in pancreatic cancer. J Hepatobiliary Pancreat Surg.

[4] Buchholz M, Biebl A, Neesse A, Wagner M, Iwamura T, Leder G (2003). SERPINE2 (protease nexin I) promotes extracellular matrix production and local invasion of pancreatic tumors in vivo. Cancer Res.

[5] Mahadevan D, Von Hoff DD (2007). Tumor-stroma interactions in pancreatic ductal adenocarcinoma. Mol Cancer Ther.

[6] Chang Q, Foltz WD, Chaudary N, Hill RP, Hedley DW (2013). Tumor-stroma inter-action in orthotopic primary pancreatic cancer xenografts during hedgehog pathway inhibition. Int J Cancer.

[7] Sato N, Maehara N, Goggins M (2004). Gene expression profiling of tumor-stromal interactions between pancreatic cancer cells and stromal fibroblasts. Cancer Res.

[8] Miyamoto H, Murakami T, Tsuchida K, Sugino H, Miyake H, Tashiro S (2004). Tumor-stroma interaction of human pancreatic cancer: acquired resistance to anticancer drugs and proliferation regulation is dependent on extracellular matrix proteins. Pancreas.

[9] Olive KP, Jacobetz MA, Davidson CJ, Gopinathan A, McIntyre D, Honess D (2009). Inhibition of Hedgehog signaling enhances delivery of chemotherapy in a mouse model of pancreatic cancer. Science.

[10] Grzesiak JJ, Ho JC, Moossa AR, Bouvet M (2007). The integrin-extracellular matrix axis in pancreatic cancer. Pancreas.

[11] Franklin O, Ohlund D, Lundin C, Oman M, Naredi P, Wang W (2015). Com-bining conventional and stroma-derived tumour markers in pancreatic ductal adenocarcinoma. Cancer Biomark.

[12] Whatcott CJ, Diep CH, Jiang P, Watanabe A, LoBello J, Sima C (2015). Des-moplasia in primary tumors and metastatic lesions of pancreatic cancer. Clin Cancer Res.

[13] Garcea G, Dennison AR, Pattenden CJ, Neal CP, Sutton CD, Berry DP (2008). Survival following curative resection for pancreatic ductal adenocarcinoma. A systematic review of the literature. JOP.

[14] Klauss M, Lemke A, Grunberg K, Simon D, Re TJ, Wente MN (2011). Intravoxel incoherent motion MRI for the differentiation between mass forming chronic pancreatitis and pancreatic carcinoma. Invest Radiol.

[15] Klauss M, Gaida MM, Lemke A, Grunberg K, Simon D, Wente MN (2013). Fib-rosis and pancreatic lesions: counterintuitive behavior of the diffusion imaging-derived structural diffusion coefficient d. Invest Radiol.

[16] Wang Y, Miller FH, Chen ZE, Merrick L, Mortele KJ, Hoff FL (2011). Diffusion-weighted MR imaging of solid and cystic lesions of the pancreas. Radiographics.

[17] Muraoka N, Uematsu H, Kimura H, Imamura Y, Fujiwara Y, Murakami M (2008). Apparent diffusion coefficient in pancreatic cancer: characterization and histopathological correlations. J Magn Reson Imaging.

[18] Legrand L, Duchatelle V, Molinie V, Boulay-Coletta I, Sibileau E, Zins M (2015). Pancreatic adenocarcinoma: mRI conspicuity and pathologic correlations. Abdom Imaging.

[19] Rosenkrantz AB, Matza BW, Sabach A, Hajdu CH, Hindman N (2013). Pancreatic cancer: lack of association between apparent diffusion coefficient values and adverse pathological features. Clin Radiol.

[20] Gaustad JV, Simonsen TG, Smistad R, Wegner CS, Andersen LM, Rofstad EK (2015). Early effects of low dose bevacizumab treatment assessed by magnetic resonance imaging. BMC Cancer.

[21] Gaustad JV, Pozdniakova V, Hompland T, Simonsen TG, Rofstad EK (2013). Magnetic resonance imaging identifies early effects of sunitinib treatment in human melanoma xenografts. J Exp Clin Cancer Res.

[22] Padhani AR, Liu G, Koh DM, Chenevert TL, Thoeny HC, Takahara T (2009). Diffusion-weighted magnetic resonance imaging as a cancer biomarker: consensus and recommendations. Neoplasia.

[23] Tofts PS (1997). Modeling tracer kinetics in dynamic Gd-DTPA MR imaging. J Magn Reson Imaging.

[24] Rofstad EK, Måseide K (1999). Radiobiological and immunohistochemical assessment of hypoxia in human melanoma xenografts: acute and chronic hypoxia in individual tumours. Int J Radiat Biol.

[25] Lyng H, Skretting A, Rofstad EK (1992). Blood flow in six human melanoma xenograft lines with different growth characteristics. Cancer Res.

[26] Vaupel PW (1994). Blood flow, oxygenation, tissue pH distribution, and bioenergetic status of tumors.

[27] Deer EL, Gonzalez-Hernandez J, Coursen JD, Shea JE, Ngatia J, Scaife CL (2010). Phenotype and genotype of pancreatic cancer cell lines. Pancreas.

[28] Tofts PS, Brix G, Buckley DL, Evelhoch JL, Henderson E, Knopp MV (1999). Estimating kinetic parameters from dynamic contrast-enhanced T1-weighted MRI of a diffusable tracer: standardized quantities and symbols. J Magn Reson Imaging.

[29] Brix G, Kiessling F, Lucht R, Darai S, Wasser K, Delorme S (2004). Microcir-culation and microvasculature in breast tumors: pharmacokinetic analysis of dynamic MR image series. Magn Reson Med.

[30] Yankeelov TE, Rooney WD, Li X, Spinger CS (2003). Variation of the relaxo-graphic “shutter-speed” for transcytolemmal water exchange affects the CR bolus-tracking curve shape. Magn Reson Med.

[31] Litjens GJS, Heisen M, Buurman J, Romeny BMTH. Pharmacokinetic models in clinical practice: what model to use for DCE-MRI of the breast? In: Proceedings of the 2010 IEEE international conference on biomedical imaging: from nano to macro, ISBI’10. New York: IEEE Press; 2010. p 185–8.

[32] Yankeelov TE, Arlinghaus LR, Li X, Gore JC (2011). The role of magnetic resonance imaging biomarkers in clinical trials of treatment response in cancer. Semin Oncol.

[33] Padhani AR (2011). Diffusion magnetic resonance imaging in cancer patient manage-ment. Semin Radiat Oncol.

[34] Lyng H, Haraldseth O, Rofstad EK (2000). Measurement of cell density and necrotic fraction in human melanoma xenografts by diffusion weighted magnetic reso-nance imaging. Magn Reson Med.

[35] Hompland T, Ellingsen C, Galappathi K, Rofstad EK (2014). Connective tissue of cervical carcinoma xenografts: associations with tumor hypoxia and interstitial fluid pressure and its assessment by DCE-MRI and DW-MRI. Acta Oncol.

[36] Barnes SL, Sorace AG, Loveless ME, Whisenant JG, Yankeelov TE (2015). Correlation of tumor characteristics derived from DCE-MRI and DW-MRI with histology in murine models of breast cancer. NMR Biomed.

[37] Mills SJ, Soh C, Rose CJ, Cheung S, Zhao S, Parker GJ (2010). Candidate bio-markers of extravascular extracellular space: a direct comparison of apparent diffusion coefficient and dynamic contrast-enhanced MR imaging-derived measurement of the volume of the extravascular extracellular space in glio-blastoma multiforme. Am J Neuroradiol.

[38] Armitage PA, Schwindack C, Bastin ME, Whittle IR (2007). Quantitative assessment of intracranial tumor response to dexamethasone using diffusion, perfusion and permeability magnetic resonance imaging. Magn Reson Imaging.

[39] Arlinghaus LR, Li X, Rahman AR, Welch EB, Xu L, Gore JC (2011). On the relationship between the apparent diffusion coefficient and extravascular extra-cellular volume fraction in human breast cancer. Magn Reson Imaging.

[40] Wang Y, Chen ZE, Nikolaidis P, McCarthy RJ, Merrick L, Sternick LA (2011). Diffusion-weighted magnetic resonance imaging of pancreatic adenocarcinomas: association with histopathology and tumor grade. J Magn Reson Imaging.

