# Supplementary material for: Diffusion-weighted and dynamic contrast-enhanced MRI of pancreatic adenocarcinoma xenografts: associations with tumor differentiation and collagen content
Source: J Transl Med. 2016 Jun 7;14:161. doi: 10.1186/s12967-016-0920-y (PMC4897888; doi:10.1186/s12967-016-0920-y)
Supplement: Supplementary file 1 — 10.1186/s12967-016-0920-y ADC did not differ with the direction of the diffusion sensitization gradient. Figure S2. Structural elements were clearly recognizable in parametric images of adjacent tumor slices. [file 12967_2016_920_MOESM1_ESM.pdf]

Additional file 1 to:

Diffusion-weighted and dynamic contrast-enhanced MRI of pancreatic adenocarcinoma xenografts: Associations with tumor differentiation and collagen content

Catherine S. Wegner[1], E-mail: Catherine.Sem.Wegner@rr-research.no

Jon-Vidar Gaustad[1], E-mail: Jon.Vidar.Gaustad@rr-research.no

Lise Mari K. Andersen[1], E-mail: Lise.Mari.Klepp.Andersen@rr-research.no

Trude G. Simonsen[1], E-mail: Trude.Golimo.Simonsen@rr-research.no

Einar K. Rofstad[1], E-mail: Einar.K.Rofstad@rr-research.no

[1]Group of Radiation Biology and Tumor Physiology, Department of Radiation Biology, Institute for Cancer Research, Oslo University Hospital, Oslo, Norway.

Correspondence: Catherine Sem Wegner, Department of Radiation Biology, Institute for Cancer Research, Norwegian Radium Hospital, Box 4953 Nydalen, 0424 Oslo, Norway. Tel: +47 2278 1223. Fax: +47 2278 1207. E-mail: Catherine.Sem.Wegner@rr-research.no

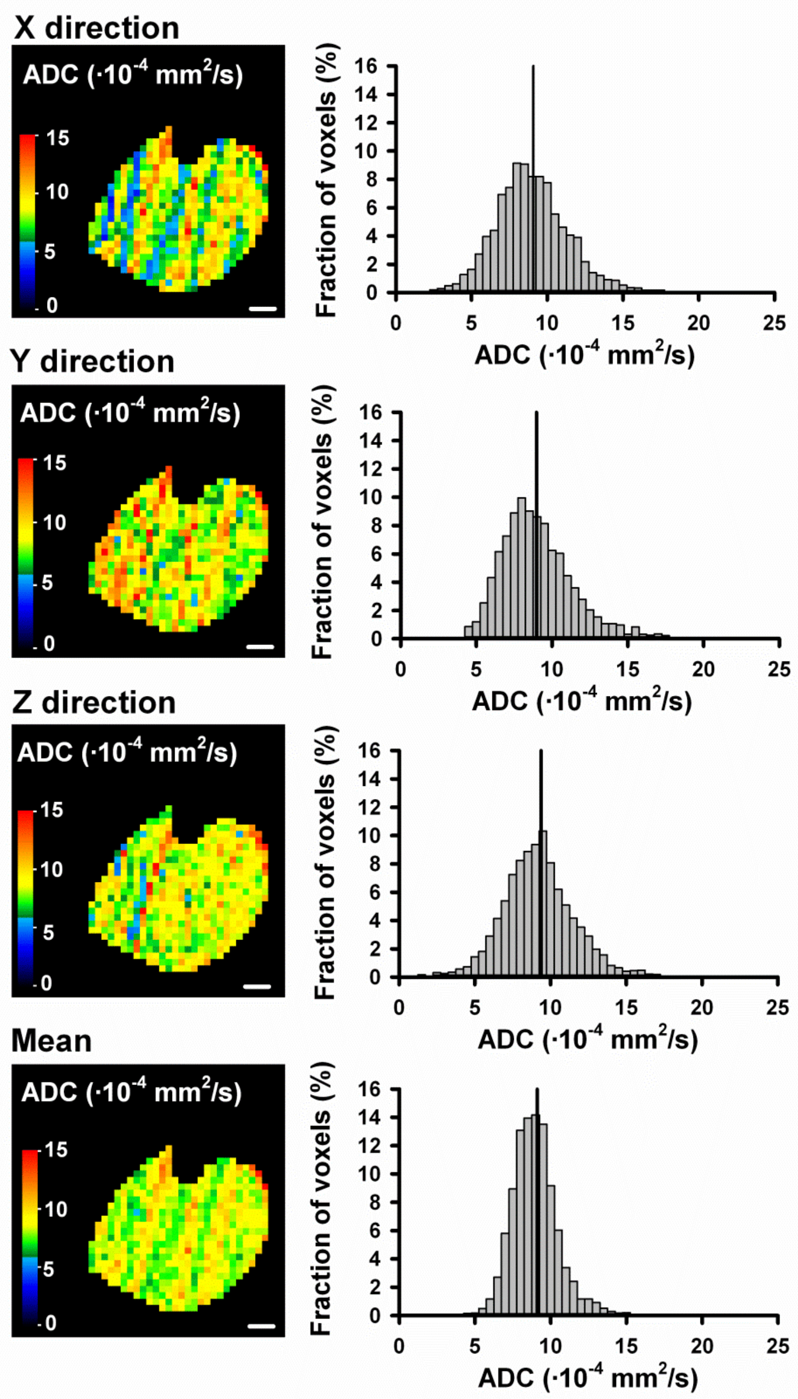

Figure S1. ADC did not differ with the direction of the diffusion sensitization gradient. ADC images and frequency distributions of a representative Capan-2 tumor obtained with the diffusion sensitization gradient in the X-direction, Y-direction, or Z-direction. The lower panels show the mean ADC image and frequency distribution calculated from the non-directional diffusion images. Color bars: ADC scale. Scale bars: 2 mm. Vertical lines: Median values.

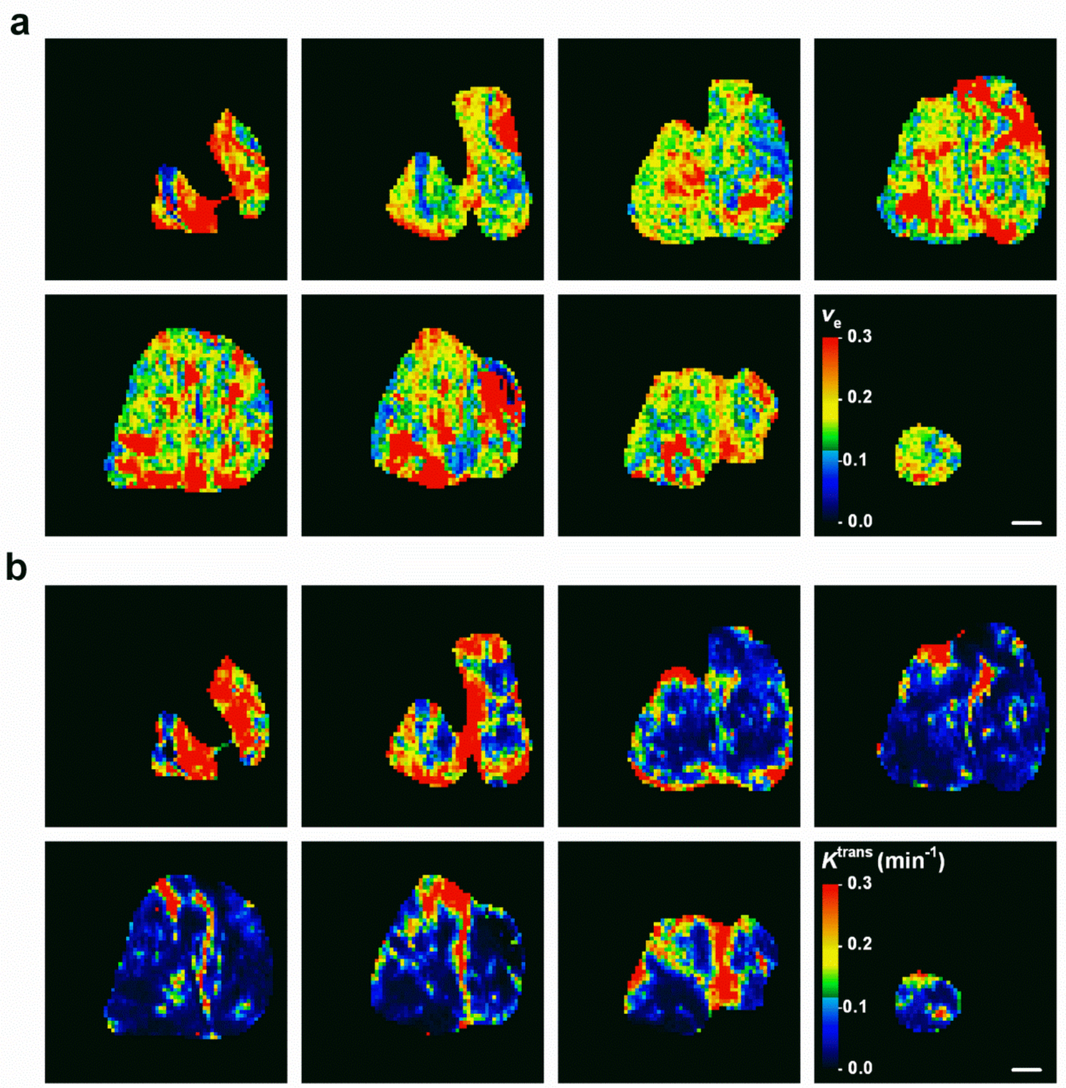

Figure S2. Structural elements were clearly recognizable in parametric images of adjacent tumor slices. Axial images of v_e (a) and K^{trans} (b) referring to eight consecutive sections through a representative Capan-2 tumor. The images are presented from the caudal end (top left) to the cranial end (bottom right) of the tumor. Color bars: v_e and K^{trans} scales. Scale bars: 2 mm.
